# A Vanishing Imprint? Modeling the Present and Future Distribution of the Enigmatic *Quercus crenata* Lam., a Mediterranean Sporadic Tree Species

**DOI:** 10.1002/ece3.71482

**Published:** 2025-05-26

**Authors:** Giuseppe Antonelli, Giuseppe Puddu, Chiara Cipollini, Gianluca Sabatini, Maurizio Conticelli, Marco Cosimo Simeone

**Affiliations:** ^1^ Department of Agricultural and Forest Sciences (DAFNE) University of Tuscia Viterbo VT Italy; ^2^ Regione Lazio – Lago di Vico Natural Reserve Caprarola VT Italy; ^3^ Fiano Romano RM Italy; ^4^ Società Cooperativa Trifolium a.r.l. Viterbo VT Italy; ^5^ Orvieto TR Italy

**Keywords:** forest biodiversity, global change, MaxEnt, Mediterranean Flora, *Quercus*, species distribution model

## Abstract

Poorly known, rare species are important biodiversity elements; understanding the relationships between their effective numbers, geographical distribution, ecology, and adaptive potential is an unquestionable critical aspect to reverse biodiversity decline under climate change. *Quercus crenata* Lam. is a sporadic Mediterranean tree species with debated taxonomy and evolutionary history. Confusing identifications and a scattered distribution combine to create an incautious lack of comprehensive and reliable information on its spatial distribution, ecology, and genetic resources, thereby hindering correct management and conservation efforts. This work undertook a first decisive step to address these knowledge gaps, integrating all previous dispersive information and presenting a comprehensive map of *Q. crenata* occurrences, with 923 established records: 495 new field observations and 428 verified from all available literature and online databases. The taxon occurs with extremely low individual numbers across central and northern Italy, southern France, western Slovenia, and Croatia, mainly at altitudes between sea level and 1100 m a.s.l. A large part of the species records are outside current networks of protected areas. The MaxEnt‐based distribution model highlights *Q. crenata* adaptation to mild Mediterranean climates with moderate temperature fluctuations, moderate‐to‐high water requirements, and diverse soil types. A broader present potential distribution than currently assessed is suggested, underlining the possibility of identifying new occurrences with accurate searches in targeted sites, thus refining the understanding of the species' actual present distribution. Future range projections under three carbon emission scenarios with increasing severity (SSP1, SSP3, and SSP5) predict substantial range losses by 2100, ranging from 32% to a drastic 99% reduction under the most severe scenario (SSP5). On these bases, our findings underline the urgent need to improve current conservation practices, which should be conveniently implemented by exhaustive genetic investigations.

## Graphical abstract

*Quercus crenata* Lam. is a sporadic Mediterranean tree species with debated taxonomy and evolutionary history. Confusing identifications and a scattered distribution combine to an incautious lack of comprehensive and reliable information on its spatial distribution, ecology, and genetic resources, thereby hindering correct management and conservation efforts. This work undertook a first decisive step to address these knowledge gaps, integrating all previous dispersive information and presenting a comprehensive map of *Q. crenata* occurrences, with 923 established records: 495 new field observations and 428 verified from all available literature and online databases.

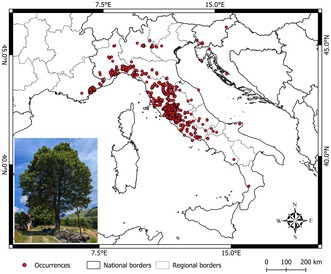

## 1 Introduction

*Quercus crenata* Lam. [= *Q*. × *hispanica* Lam.; *Quercus* (Fagaceae), subgen. *Cerris*, Sect. *Cerris* (Denk et al. 2017)] is a sporadic tree species of the South‐central Mediterranean region that has long captivated the attention of researchers due to its iconic appearance, controversial taxonomy, enigmatic distribution, and recently questioned evolutionary history.

It is a large (up to 2 m of diameter and over 20 m height), spreading tree, with crenate leaves that are shiny green above and whitish pubescent below, and large acorns (maturing in 2‐years) with slender, non‐appressed cupscales (Pignatti et al. 2017). The silvery corky bark (1–2 cm thick) and the semievergreen habit, together with its architecture and longevity, are probably the main features making this tree particularly attractive to the point that many plants are presumed to have been intentionally dispersed (or preserved) by man as symbols of the local natural and cultural heritage (Armiraglio et al. 2003). Many majestic plants today stand in numerous arboreta, parks, and botanical gardens in Central Europe (https://www.internationaloaksociety.org/). Although widely adopted by the nursery trade in the form of numerous vegetatively propagated cultivars (https://www.treesandshrubsonline.org/), only a few hundred plants are known to thrive in the wild as sporadic members of mixed deciduous oak forests and, quite frequently, isolated trees in forest margins along roadways, private gardens, edges, and open spaces in country contexts (Grossoni et al. 2020).

Known in historic floras since antiquity (Cristofolini et al. 2017), the taxonomy of *Q. crenata* , first formally described by Lamarck (1785), has been the subject of considerable debate, adding several alternative names to a rather complex nomenclature (reviewed in Cristofolini et al. 2017). Although generally considered a natural hybrid between *Q. cerris* and *Q. suber* (Schwarz 1936–1939; Pignatti et al. 2017), two oaks with partially overlapping distributions, its occurrence in regions lacking *Q. suber* and recent phylogenomic data (Hipp et al. 2020; Denk et al. 2023) challenge the simple hybridization hypothesis, revealing a more intricate evolutionary history. In particular, Denk et al. (2023) inferred a dated phylogeny of *Quercus* Sect. *Cerris* using Restriction site‐Associated DNA sequencing (RAD‐seq) and a fossilized birth–death (FBD) model calibrated with 47 fossils. Unexpectedly, the obtained phylogenomic tree resolved *Q. crenata* as a less‐derived species, likely differentiated ca. 10–5 Ma (Late Miocene to Early Pliocene), within an autonomous evolutionary lineage (subsection *Suber*) that includes *Q. suber* and diverged from the remaining Western Eurasian members of the same section (including *Q. cerris* ) ca. 24 Ma (Late Oligocene to Early Miocene). Denk et al. (2023) also tested the introgressive hybridization hypothesis with the Patterson's D‐statistic test (Durand et al. 2011) and recorded consistent support for admixture with *Q. cerris* as well as with other oak species within the same section, suggesting ancient introgression among precursors of the modern species and retention of ancient polymorphisms as likely explanations. In agreement with previous nuclear ribosomal DNA sequence data (Simeone et al. 2018; see also Bellarosa et al. 2005) and further supported by several symplesiomorphic traits of the leaf morphotype (e.g., the strongly developed teeth with mucronate to cuspidate tips), and other putatively ancestral traits within Sect. *Cerris* (including the corky bark and semievergreen habit), new hypotheses on the origins of *Q. crenata* were thus advanced: the species may represent the remainder of an ancestral form rather than the product of secondary contacts between *Q. suber* and *Q. cerris* , as traditionally assumed based on a mere phenotypic intermediacy.

Although based on low numbers (< 10 individuals analyzed), these findings contribute to solidify “ *Q. crenata* ” as the correct and valid name, stabilize its classification, and point to a remarkable importance as a relict species among West Eurasian oaks. Nevertheless, the coexistence of *Q. crenata* with hybrid/introgressed *Q. cerris* × *Q. suber* individuals is feasible (Cristofolini and Crema 2005; Conte et al. 2007; Simeone et al. 2018). The two forms are morphologically, genetically, and ecologically hardly traceable, further complicating a reliable comprehension of the true nature of this *taxon*. An adequate assessment of the species' occurrence would certainly assist larger samplings and deeper studies to definitely assess whether we should consider *Q. crenata* as an irreplaceable evolutionary leftover within West Eurasian oaks or as an exemplary model of hybridization/introgression, possibly facilitating adaptation, migration, and demographic resilience (Petit et al. 2004; Dodd and Afzal‐Rafii 2004).

Nonetheless, the sporadic nature and complex taxonomy of *Q. crenata* have contributed to a significant uncertainty regarding its distribution. Current knowledge generally reports sparse occurrences extending from central Italy to southern France, Slovenia, and Croatia (Jalas and Suominen 1976). Further (historical) records are disputed, particularly those from the Iberian Peninsula, Albania, Montenegro, and Sicily, where the *taxon* identity and presence are considered either uncertain or non‐native (Amaral Franco 1990; Cristofolini et al. 2017; Barina et al. 2018). In Italy, preliminary assessments report *Q. crenata* at approximately 200 sites, concentrated along the central and northern Tyrrhenian coast of Italy, with scattered occurrence in the central Apennines and Prealps, and very limited information for the Adriatic coast and southern Italy (Mercurio 1985; Cresta and Salvidio 1991; Armiraglio et al. 2003; Conte et al. 2007). Today, *Q. crenata* is considered “not evaluated” in the Red List of Oaks (Eastwood and Oldfield 2007), and “Data Deficient” in the IUCN Red List of the European Trees (Rivers et al. 2019). Today, *Q. crenata* is considered “not evaluated” in the Red List of Oaks (Eastwood and Oldfield 2007), and “Data Deficient” in the IUCN Red List of the European Trees (Rivers et al. 2019). It is, however, listed as “Vulnerable” in the National Red Lists of vascular flora of France (UICN France 2018), cited as “Endangered” in the Slovenian report of the Global Forest Resources Assessment 2005 (FAO 2005) and is under protection by law in seven Italian administrative regions.

Understanding the relationships between species' geographical distribution and climatic factors is a critical ecological aspect for biodiversity conservation, especially under the current climate change (Pecl et al. 2017; Adam et al. 2022). In this view, poorly known species with small geographical ranges and low numbers of individuals should be given high conservation priority because they might face a high extinction risk due to demographic, genetic and ecological stochasticity (Vincent et al. 2020). Ideally, sound assessment of a species' population counts, distribution, and ecology would allow evaluating the impact of climate change and the consequent application of efficient conservation strategies. Recent research with Species Distribution Models (SDMs) has demonstrated that climate warming will significantly reduce and reshape the geographical distribution of many European tree species (Dyderski et al. 2025), including oaks (Vessella et al. 2017; Suicmez and Avci 2023; Özcan et al. 2024; Sadeghi et al. 2024). Because of the absence of a thorough study, *Q. crenata* is lagging behind in knowledge and detailed analyses, with only a handful of surveys focusing on local and regional territories within its range.

SDMs predict the geographic distribution of species by analyzing the relationship between known occurrences and environmental variables (Franklin 2010). These models generate probabilities that reflect the likelihood of a species' presence in a given area, identifying areas with similar environmental conditions. These probabilities can be interpreted as relative habitat suitability (Guisan et al. 2017), providing insights into current distributions, potential range shifts under climate change, and informing conservation planning. MaxEnt (Maximum Entropy; Phillips et al. 2006) is a powerful machine learning tool well‐suited for species with sparse and uneven occurrence data, especially those with limited presence‐only records (e.g., Gao et al. 2022; Zhu et al. 2024; Dong et al. 2025). In this study, we developed a statistically robust MaxEnt‐based SDM to model the current and future distribution of *Q. crenata* using verified and operable occurrence data. Our objectives were to: (a) provide an overdue exhaustive assessment of *Q. crenata* 's actual distribution, evaluating whether its sporadic nature might entail rarity; (b) determine the primary environmental factors influencing its potential distribution, highlighting regions where the species may have been overlooked; and (c) predict its vulnerability under future climatic conditions. The findings will contribute to a better understanding of the ecology of *Q. crenata* , recommend priority areas for its effective conservation, and serve as a baseline for future field searches and further investigations on its genetic diversity.

## 2 Materials and Methods

### 2.1 Presence Data and Study Area

All *Q. crenata* presence data were compiled from georeferenced records. The primary data source were field surveys conducted by the authors during the winter seasons from 2008 to 2024 in central Italy. Scientific publications and gray literature (including technical reports, policy and working documents, newsletters) documenting *Q. crenata* punctual occurrence and unequivocal identity all over its range were critically reviewed and supplemented with free contributions from botanists and forestry professionals. The dataset was further enriched with verified data from publicly accessible online databases (Alfeus et al. 2025), including iNaturalist (https://www.inaturalist.org/), GBIF (https://www.gbif.org/), Wikiplantbase #Italia (https://bot.biologia.unipi.it/wpb/italia), and the French National Inventory of Natural Heritage (https://inpn.mnhn.fr/). Due to the heterogeneity of the collected presence data, each report was treated as a unique occurrence.

To assess the conservation status of *Q. crenata* , we overlaid its occurrence records with spatial data on Natura 2000 sites (retrieved from https://www.eea.europa.eu/data‐and‐maps/data) and on national or regional official documentation where the species is under legal protection.

The study area for the distribution model includes all regions with verified occurrences of *Q. crenata* , namely peninsular Italy, southern France, Slovenia, and coastal Croatia. To account for historical range dynamics and potential future shifts (Barve et al. 2011), we expanded the study area to include the rest of the French Mediterranean coast, parts of the Catalonian coast, sections of inland France and Switzerland, the Balkan coasts extending to Albania, and parts of the inland Balkans, as well as Sicily, Sardinia, Corsica, and smaller islands. This expanded area spans a total of 811,900 km^2^.

To mitigate potential biases in the spatial distribution of *Q. crenata* , which could have led to an over‐representation of specific environmental parameters in the model, we implemented a standardized sampling approach: the study area was divided into 1 km^2^ grid cells using the WGS 84/UTM 32N (EPSG:32632) coordinate system, and the centroid of each grid cell containing at least one occurrence was retained, thus ensuring only a single representative location per cell. The final number of unique occurrence points retained for modeling is 705.

### 2.2 Environmental Data

A combination of bioclimatic, soil, and topographic data was employed to describe the environmental niche and the current potential distribution of *Q. crenata* .

Bioclimatic variables representing present conditions were sourced from the CHELSA repository (Karger et al. 2017), version 2.1 (Karger et al. 2021). They consist of nineteen bioclimatic variables, recognized as key drivers of species distributions computed over the period 1981–2010, with a native resolution of 30 arc‐sec.

Soil data, extracted from the Harmonized World Soil Database v2.01 (FAO and IIASA 2023), also had a native resolution of 30 arc‐sec. The selected layer classifies soils according to the World Reference Base for Soil Resources (IUSS Working Group WRB 2022), providing a broad yet ecologically meaningful summary of edaphic conditions relevant to the species' distribution at the resolution of our study.

Topographic data, including elevation, slope, and aspect, were obtained from ESA Copernicus GLO‐90 v2023.1 (European Space Agency 2024), with a finer native resolution of 90 m.

All these raster layers were resampled to a common resolution of 1 km using the study area grid as a reference. Their descriptions are reported in Appendix A: Table A1.

To address potential multicollinearity, a statistical issue that arises when independent variables in a regression model are highly correlated, we conducted an iterative variable selection process using Pearson's correlation coefficient. A correlation matrix was calculated for all 23 environmental variables (3 topographic, 1 soil, and 19 bioclimatic) and pairs of variables with a correlation coefficient (|*r*|) greater than 0.7 (Dormann et al. 2013) were identified as highly correlated. To remove redundancy, we iteratively removed the variable with the highest average correlation to the others, recalculated the correlation matrix, and repeated the process until all remaining variables had a correlation coefficient below the threshold. Variables BIO12 (annual precipitation), BIO4 (temperature seasonality), and BIO15 (precipitation seasonality) were prioritized in this process; due to their established ecological importance, they were retained, and the algorithm focused on removing variables that were highly correlated with them. In particular, BIO12 was included as it provides a fundamental measure of precipitation, capturing the overall influence of precipitation without bias toward specific ecological hypotheses, while BIO4 and BIO15 were retained for their value in understanding climatic patterns, as they help differentiate between Mediterranean and temperate climates. The resultant list of variables thus incorporates these three retained variables in addition to the remaining variables chosen by the iterative variable selection process.

Additionally, to further assess potential multicollinearity among the resultant variables, a Variance Inflation Factor (VIF) analysis was performed as a secondary check, as Pearson's correlation analysis may not always identify complex forms of multicollinearity; a threshold of 5 was used, where values below 5 indicate low correlation among predictors (James et al. 2013).

### 2.3 MaxEnt Modeling

The distribution of *Q. crenata* was modeled using MaxEnt version 3.4.4 (Phillips et al. 2006) within an R environment. Given the unplanned sampling and heterogeneous distribution of presence data, MaxEnt was a suitable choice for its robustness in handling such data (Phillips and Elith 2013).

To optimize MaxEnt model parameters and reduce overfitting, 50 unique combinations of feature classes and regularization multipliers were tested. The feature classes tested included linear (L), quadratic (Q), product (P), hinge (H), and threshold (T), in the following configurations: L, LQ, LQH, LQHP, LQHPT; regularization multipliers ranged from 0.5 to 5.0 in 0.5 increments. All models were run using 20,000 background points.

To account for spatial autocorrelation, we employed a block cross‐validation approach, dividing the study area into four spatially distinct blocks; this ensured independence between training and testing data by preventing the random assignment of occurrences and background points. By leveraging data heterogeneity, this approach minimizes spatial autocorrelation, making it particularly suitable for model transfer across space or time (Wenger and Olden 2012; Radosavljevic and Anderson 2014). For each iteration, the model was trained on three blocks and tested on the remaining one.

Different metrics were assessed to choose the best‐performing model, specifically: Akaike Information Criterion correction (AICc; Burnham and Anderson 1998) was used for model selection, balancing model fit with complexity; the absolute difference between training and validation AUC (AUC_diff_; Warren and Seifert 2011) was used to detect overfitting, with smaller differences indicating better generalization; and the Continuous Boyce Index (CBI; Hirzel et al. 2006), particularly useful for presence‐only data, was used to assess the agreement between predicted and observed distributions, providing insight into the model's ability to capture ecological patterns. Finally, the response curves of the best‐performing combinations were examined to select the most ecologically interpretable model. These curves visualize how the predicted probability of occurrence changes with environmental conditions, providing insights into the species' niche (Elith et al. 2006). This visualization allowed us to assess the plausibility of the model's predictions and directly inform the selection of the model that best balanced high predictive performance, low overfitting, and ecological relevance.

A detailed description of the overall modeling workflow including Overview, Data, Model, Assessment, and Prediction (ODMAP protocol; Zurell et al. 2020) can be found in Appendix A: Table A2.

### 2.4 Potential Distribution Analysis

To better understand the spatial variation in the probability of *Q. crenata* occurrence, we categorized the predicted probabilities into four classes. These classes were defined using the following thresholds: *Minimum Training Presence* (MTP), the lowest probability at which the model correctly predicted the species' presence during training; *10th Percentile Training Presence* (P10), representing the probability below which 10% of the training presence points fell; and a fixed threshold of 1–1/e ≈ 0.632, corresponding to a predicted abundance of one individual per cell in a *cloglog* output (Phillips et al. 2017).

### 2.5 Future Projections

To project future distributions of *Q. crenata* , we considered three time periods: 2011–2040, 2041–2070, and 2071–2100. Future bioclimatic data for these periods were also obtained from the CHELSA repository to maintain consistency with the climatic layers used for present‐day distribution modeling. Specifically, these data were derived from the Max Planck Institute Earth System Model (MPI‐ESM1.2) for the High‐Resolution Model Intercomparison Project (HighResMIP) hereafter referred to as MPI‐ESM1.2‐HR (Gutjahr et al. 2019) and describe global climate changes under three Shared Socioeconomic Pathways (SSP; IPCC 2021) scenarios: SSP1‐2.6 (optimistic), SSP3‐7.0 (moderate), and SSP5‐8.5 (pessimistic). The layers were obtained from the CHELSA repository at a downscaled resolution of 30 arc‐sec and were subsequently resampled to 1 km resolution.

Our selection of MPI‐ESM1.2‐HR was driven by its high native resolution, the highest among the ESMs available within the CHELSA repository (Karger et al. 2021). This factor is particularly advantageous for the Mediterranean region, characterized by its complex and fine‐scale climatic heterogeneity, enabling a better representation of relevant physical processes and potentially reducing biases in atmospheric and oceanic dynamics. Considering this is the first comprehensive assessment of *Q. crenata* distribution, and our primary focus on establishing a fundamental understanding of its present distribution, we opted for this single, high‐resolution model to provide an initial yet valuable insight into the species' vulnerability to future climate change.

Given the relatively slow rates of change in topographic and pedological features, these variables were assumed to remain constant in future projections.

To better visualize the areas in which *Q. crenata* could be present in the future, and to delineate possible conservation areas, in the future projection maps the predicted probability was categorized into only two classes: above and below the fixed threshold of 0.632, representing high and low suitability, respectively. We quantified the number of raster cells with high suitability and computed the percentage difference between current and future cells across all occurrence probability classes.

The reliability of both present and future projections was assessed using a Multivariate Environmental Similarity Surface (MESS) analysis (Elith et al. 2010), a method that compares the environmental conditions of the projected areas to those used in model training, thereby highlighting areas of dissimilarity.

## 3 Results

### 3.1 Updated Map of *Q. crenata* Distribution

**FIGURE 1:**
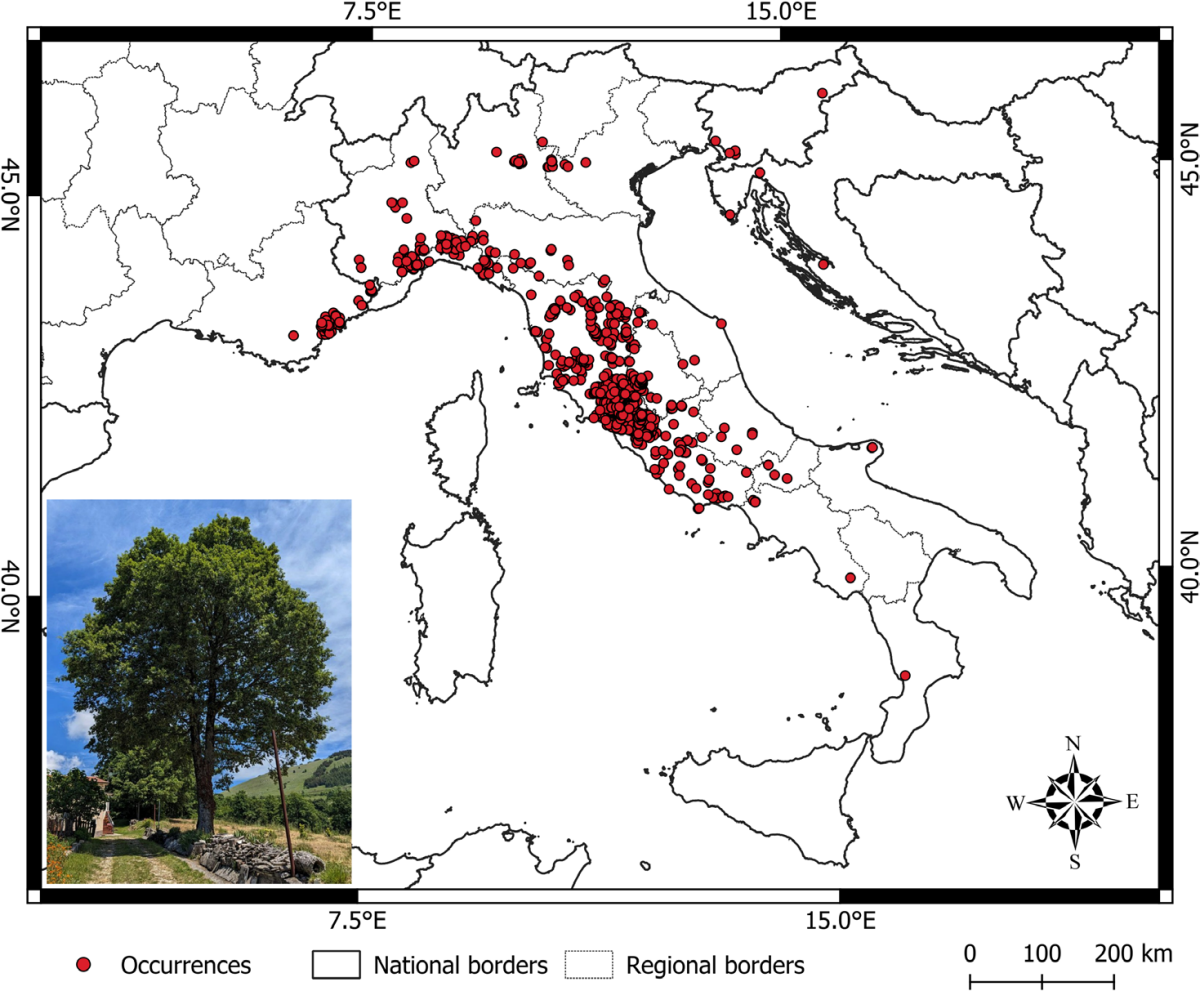
Collected occurrence of *Quercus crenata* . “Regional Borders” refer to the first‐level administrative regions in both France and Italy. National and Regional borders source: https://earthworks.stanford.edu/.

The data collection process resulted in a total of 923 verified and georeferenced occurrences of *Q. crenata* (full dataset description available at: https://zenodo.org/records/14945096). These included 480 records obtained through the authors' field surveys, 213 extracted directly from scientific and gray literature, 215 sourced from online databases, and 15 contributed by forestry professionals and botanists. The total number of the occurrences is the result of a selection process aimed at removing duplicates whenever the available information was sufficient, such as when the location was clearly repeated, and form the basis for the first comprehensive map of *Q. crenata* actual distribution (Figure 1). Combined, the 495 newly field‐observed and contributed records (53.6% of total) represent fresh data that integrate previously dispersive information and improve the knowledge on the present species' range.

This total number of occurrences is the result of a selection process aimed at removing duplicates whenever the available information was sufficient, such as when the location was clearly repeated.

Overall, the regions along the Tyrrhenian and Ligurian‐Provençal Sea coast collectively exhibited the highest number of recorded occurrences, totaling 647 observations: 430 in Latium (West‐central Italy), 218 in Tuscany (West‐central Italy), 58 in Liguria (West‐northern Italy), and 48 in Provence‐Alpes‐Côte d'Azur (South France). Significant occurrences were also observed across the entire arc of the Italian Prealps, whereas only scattered specimens were found on the eastern Apennines slopes, in Southern Italy, and along the Italian, Slovenian, and Croatian Adriatic coasts. Occurrences were recorded across a broad elevational range, from 1 to 1086 m a.s.l. with a mean of 473 m a.s.l., while isolated occurrences were observed up to 1623 m a.s.l. Many individuals appeared to be in a state of vegetative health, and seed production was reported in many cases, although its extent varied. However, limited natural regeneration was evidenced in stands mostly located in the Central (Lazio, Tuscany) and Northern (Liguria, Piedmont) Italian regions.

Additional 136 occurrences, mostly collected in historic herbaria and online databases, were not included in the analyses due to a lack of precise location data and/or insufficient reliability. Overall, the excluded records did not provide additional geographic information to the described distribution pattern, since the largest part of these records were from areas largely well‐covered by georeferenced data. They, however, also included a small number of interesting records (nine in total) from Southern Italy (Basilicata and Calabria), the Italian Adriatic coast (Abruzzo), and the Southern French coast (Occitanie), which are regions where records of the species are comparatively scarce (see dataset description: https://zenodo.org/records/14945096).

### 3.2 Environmental Variable Selection and Model Performance

After filtering the data to ensure unique occurrences within 1 km^2^ grid cells, 705 records were used to model the taxon's potential distribution.

**FIGURE A1:**
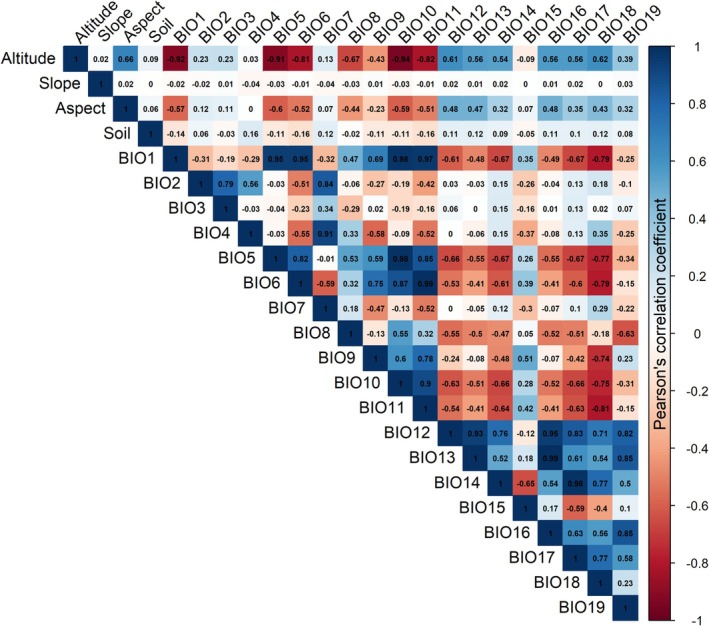
Correlation matrix of all 23 environmental variables (3 topographic, 1 soil, and 19 bioclimatic) showing Pearson's correlation coefficients (*r*).

**FIGURE A2:**
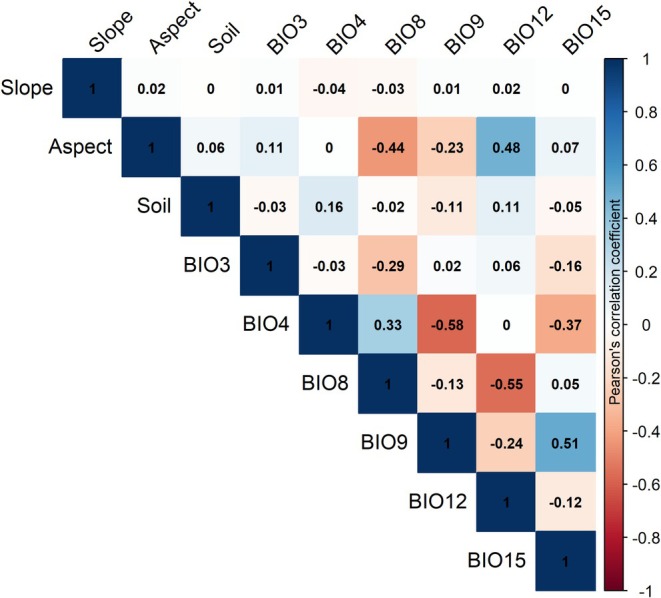
Correlation matrix of the nine remaining environmental variables after iterative removal of highly correlated variables (|*r*| < 0.7).

**FIGURE A3:**
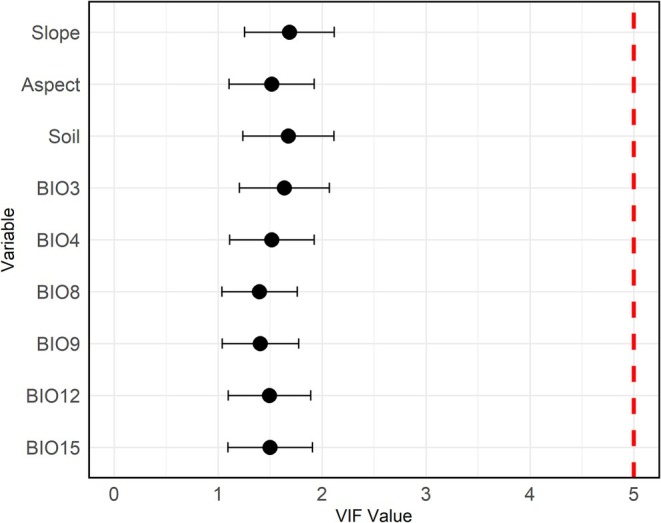
Variance Inflation Factor (VIF) analysis. All variables exhibit VIF values less than 5, indicating low collinearity.

The iterative selection process used to address potential multicollinearity identified a final set of nine variables: slope, aspect, soil type, and six bioclimatic variables (BIO3, BIO4, BIO8, BIO9, BIO12, BIO15; see Appendix A: Figures A1 and A2). This reduced set effectively represents the environmental conditions of the study area while minimizing redundancy. Moreover, selected variables had a maximum VIF value of 2.2, indicating that multicollinearity was adequately mitigated (see Appendix A: Figure A3).

The results from the evaluation of the 50 candidate models showed that the LQHPT models outperformed others in terms of AICc (see Appendix A: Table A3). However, after analyzing the response curves, the LQHPT models displayed irregular, step‐like patterns that lacked ecological interpretability. LQHP models, on the other hand, while performing well in terms of AICc, exhibited smoother, more biologically plausible response curves, which facilitated clearer ecological interpretation and a better understanding of the species' environmental preferences. The best‐performing LQHP model in terms of AUC_diff_ and CBI, selected for subsequent SDM development, was the one with a regularization multiplier of 2, showing an AUC_diff_ of 0.047 ± 0.032 (the lower the better) and an average CBI on the validation data of 0.876 ± 0.140 (maximum = 1).

### 3.3 Modeled Environmental Niche of *Q. crenata*

The most influential variables contributing to the MaxEnt model (Table 1) were precipitation seasonality (BIO15), which had the highest contribution at 32.3%, followed by temperature seasonality (BIO4) at 22.9%, and annual precipitation (BIO12) contributing 14.9%. Pedological factors (Soil) and mean daily temperature of the driest quarter (BIO9) contributed similarly, with 10.2% and 10.1%, respectively; however, BIO9 exhibited a higher permutation importance (10.4%) compared to Soil (3.6%), indicating that BIO9 had a stronger influence on model performance.

**TABLE 1:** Percentage contribution and permutation importance of environmental variables included in the Maxent model to predict the potential distribution of *Quercus crenata*.

Variable	Description	Percent contribution	Permutation importance
BIO15	Precipitation seasonality	32.3	40.7
BIO4	Temperature seasonality	22.9	22.3
BIO12	Annual precipitation amount	14.9	9.6
Soil	WRB soil classification classes	10.2	3.6
BIO9	Mean daily mean air temperatures of the driest quarter	10.1	10.4
BIO8	Mean daily mean air temperatures of the wettest quarter	3.5	5.3
BIO3	Isothermality	3.5	4.6
Slope	Slope	2.2	3.3
Aspect	Aspect	0.5	0.3

**FIGURE 2:**
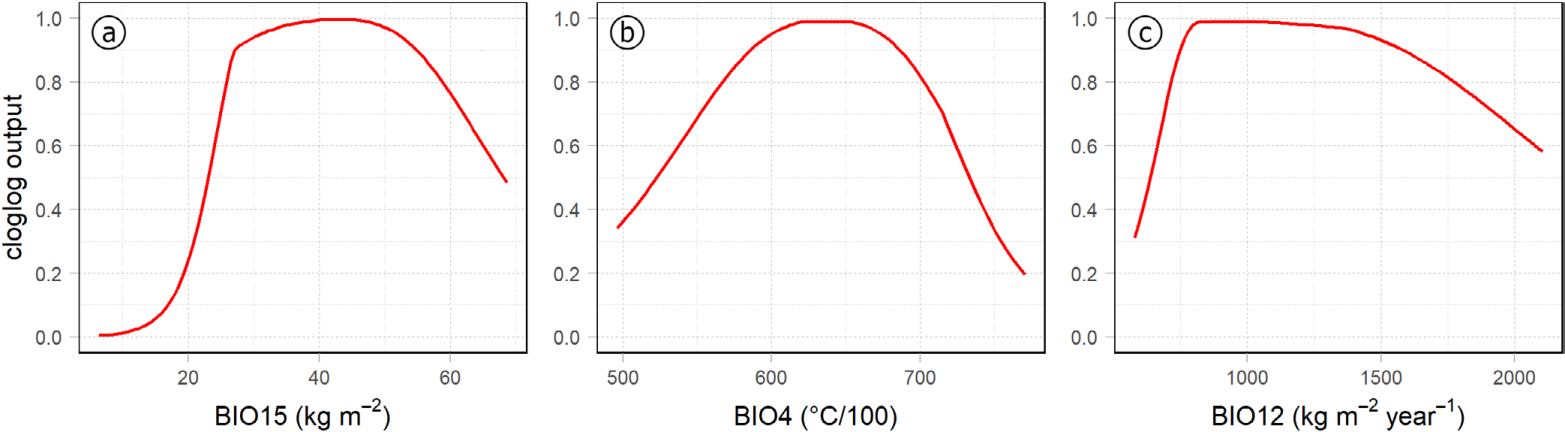
Response curves of the three most influential variables (based on permutation importance): (a) BIO15 (precipitation seasonality), (b) BIO4 (temperature seasonality), and (c) BIO12 (annual precipitation amount).

The response curves of the three most contributing variables (Figure 2) illustrate how the species' probability of presence changes with each environmental variable, while the variables are kept constant at their mean values. For precipitation seasonality (BIO15), a unimodal relationship is observed, with peak suitability around 40 and 50 mm/year, indicating a preference for Mediterranean climatic conditions. Temperature seasonality (BIO4) shows a similar trend, with an optimum between 610 and 660 (6.1°C and 6.6°C), suggesting a preference for a mild Mediterranean climate with moderate temperature fluctuations. BIO12 (annual precipitation) exhibits a maximum suitability for values between 650 and 1100 mm while showing a slightly asymmetrical shape, suggesting that the species has moderate‐to‐high water requirements and is not well adapted to drought. Regarding the edaphic conditions, *Q. crenata* looks adaptable to different soil types.

**FIGURE A4:**
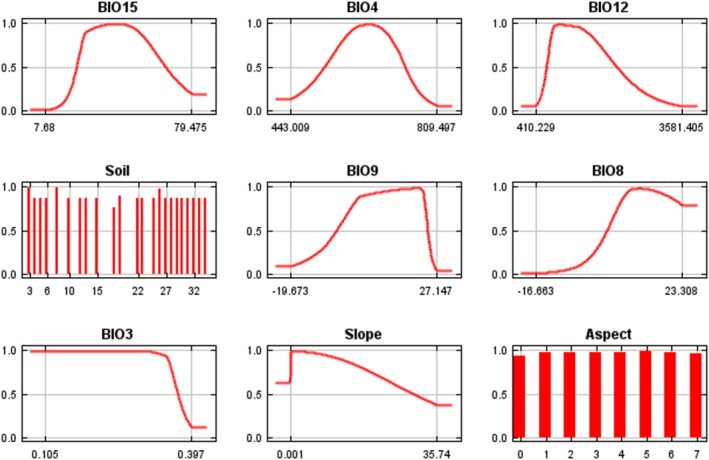
Response curves showing the effect of each environmental variable on predicted probability of species presence while all other variables were held at their mean value.

All obtained response curves are reported in Appendix A: Figure A4.

### 3.4 Predicted Current Potential Distribution

Based on the model, the thresholds chosen to better visualize the spatial variation in the probability of *Q. crenata* occurrence were as follows: 0.0078 for Minimum Training Presence and 0.1746 for the 10th Percentile Training Presence, in addition to 0.632 as a fixed threshold. Probability classes were then defined as follows: High (values exceeding 0.632), Moderate (0.632 to 10P), Low (10P to MTP), and Negligible (values below the MTP).

**FIGURE 3:**
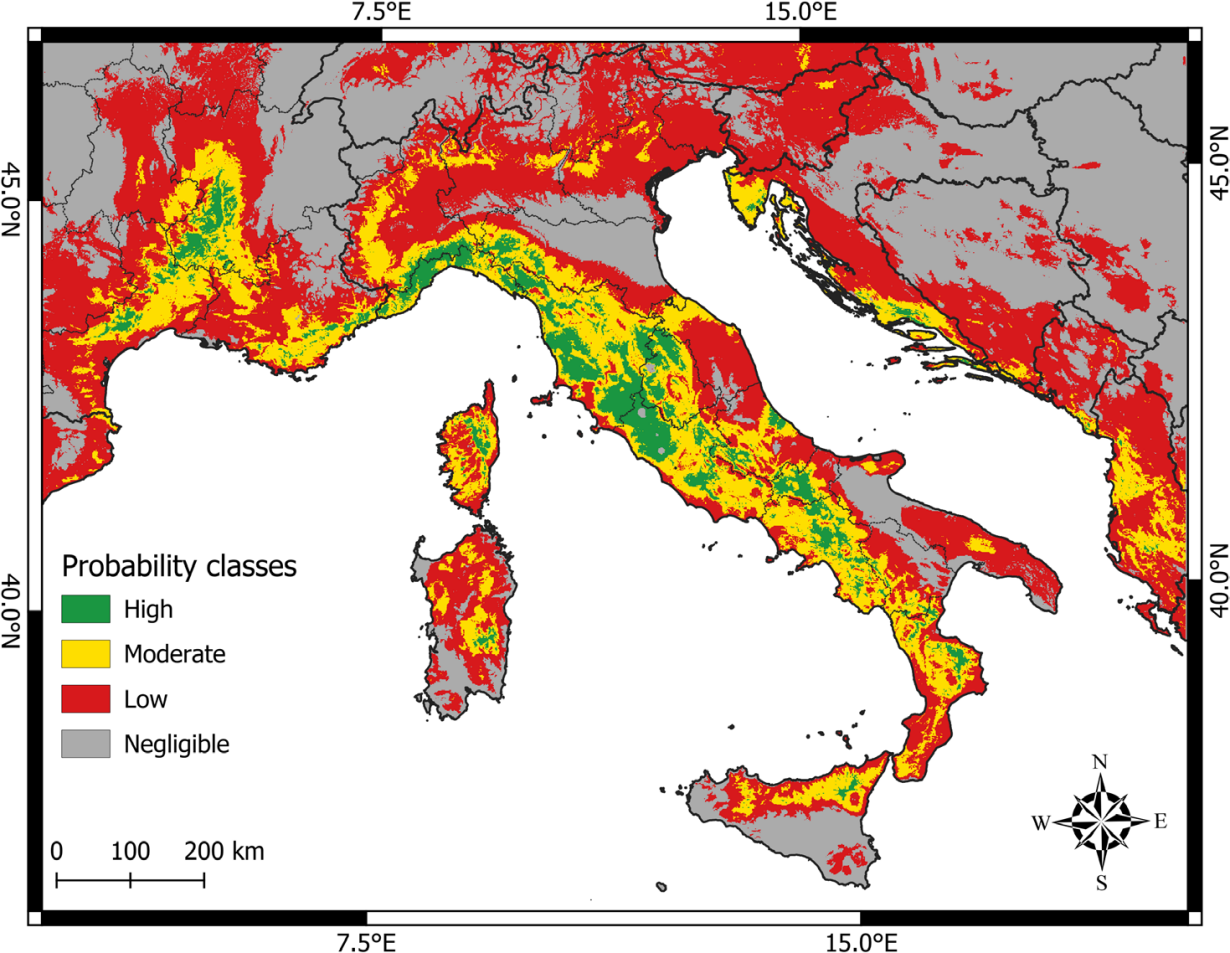
Map of the current potential distribution of *Quercus crenata* . Probability classes are defined as follows: High for values exceeding 0.632; moderate values from 0.632 to 0.1746 (10P); low values from 0.1746 (10P) to 0.0078 (MTP); negligible values below 0.0078 (MTP).

The areas resulting with high probability of presence (or high suitability, Figure 3) extend across central and northern Italy, particularly in well‐documented areas such as northern Latium, west Umbria, Tuscany, and Liguria, highlighting a continuity of suitable habitats stretching up to the French Riviera in southern France. In Italy, more restricted and fragmented areas with high suitability were also detected in regions with fewer documented occurrences, such as coastal areas in central Abruzzo, Samnite and Campanian Apennines (Molise and Campania) and Pollino and Sila massifs in Calabria. In Croatia, areas of high suitability are scattered along the coast, with limited overlap with the locations of the recorded occurrences. In France, besides the well‐documented Provence‐Alpes‐Côte d'Azur, high suitability was also predicted in eastern Occitanie and southern Auvergne Rhône‐Alpes regions, particularly in the Rhône River basin, where no occurrences have been recorded. Other areas, including Sicily, Sardinia, Corsica, and parts of Albania, also exhibited scattered high‐suitability areas despite the lack of any historical or current documented occurrence. The total extent covered by high‐suitability areas resulted in 35,273 km^2^, corresponding to 4.4% of the study area extent (Table 2).

**TABLE 2:** Total extent of present projection probability classes.

Probability class	Thresholds	Area	% of the study area
High	Above 0.632	35,273 km^2^	4.4%
Moderate	0.1746 to 0.632	126,406 km^2^	15.5%
Low	0.0078 to 0.1746	330,717 km^2^	40.6%
Negligible	Below 0.0078	320,777 km^2^	39.5%

Areas predicted to have moderate probability of presence, or moderate suitability, often border regions with high suitability. However, some areas of moderate suitability were also identified in disjunct locations, such as across the Italian Prealps up to Slovenia, where numerous occurrences were documented, and along the Adriatic coast in northern Marche, and in Gargano Promontory and Murge Plateau in Apulia, where only a few sparse occurrences were found.

### 3.5 The Impact of Climate Change on the Distribution of *Q. crenata*

**FIGURE 4:**
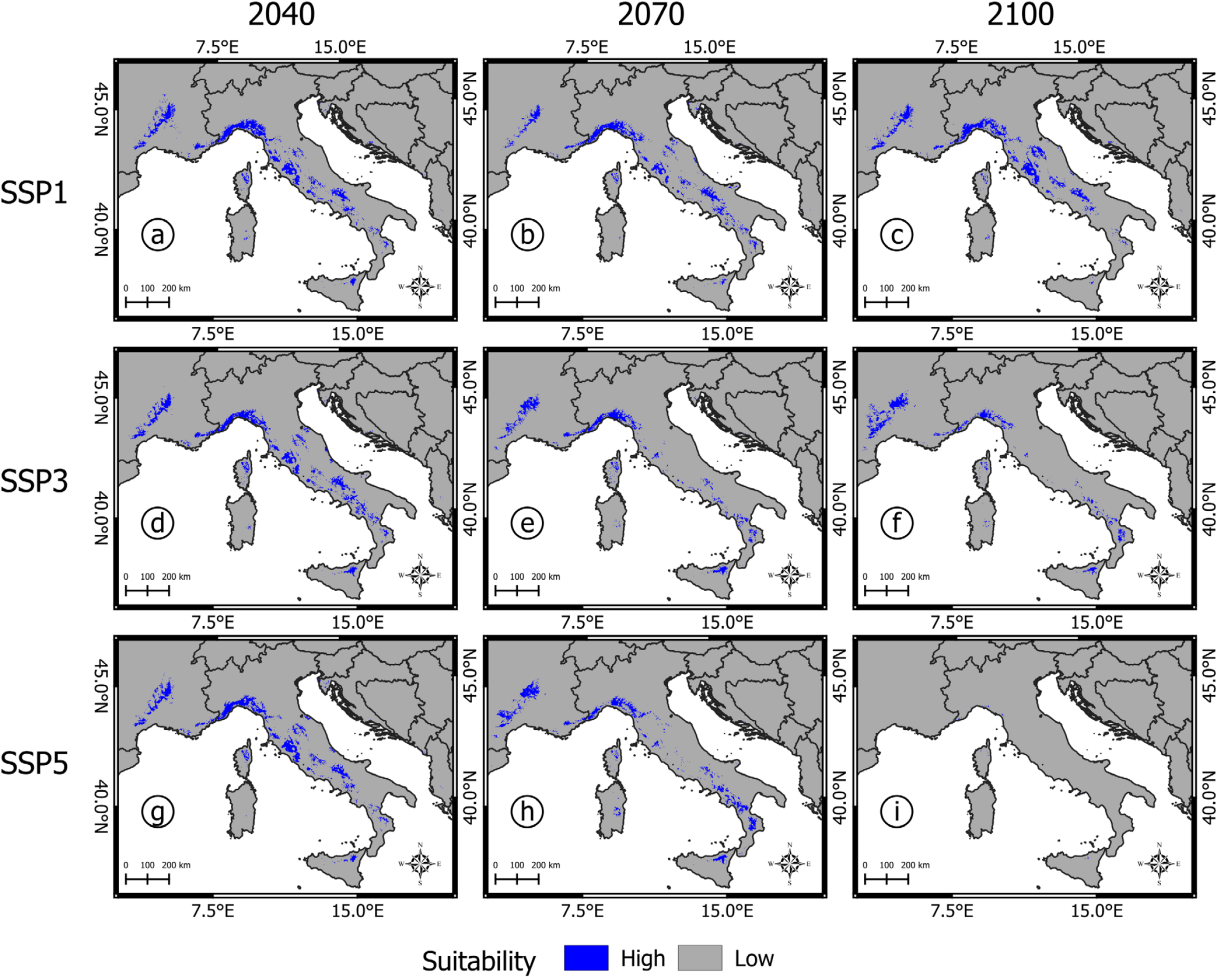
Future projections of *Quercus crenata* high‐ and low‐suitability areas under different future scenarios: (a) by 2040 under SSP1; (b) 2070 SSP1; (c) 2100 SSP1; (d) 2040 SSP3; (e) 2070 SSP3; (f) 2100 SSP3; (g) 2040 SSP5; (h) 2070 SSP5; (i) 2100 SSP5.

**FIGURE 5:**
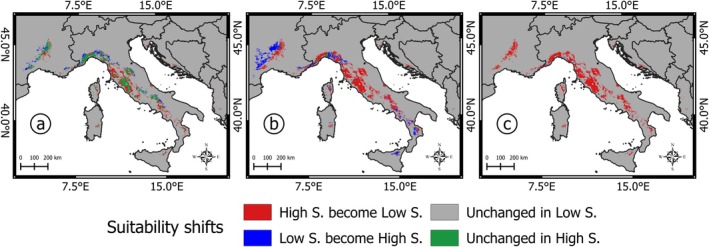
Comparison between the current and future (by 2100) high‐ and low‐suitability areas under different scenarios: (a) SSP1; (b) SSP3 (b); (c) SSP5.

Projections for *Q. crenata* under three SSP scenarios (SSP1‐2.6, SSP3‐7.0, and SSP5‐8.5) reveal different trajectories in habitat suitability over the 21st century (Figures 4 and 5).

**FIGURE 6:**
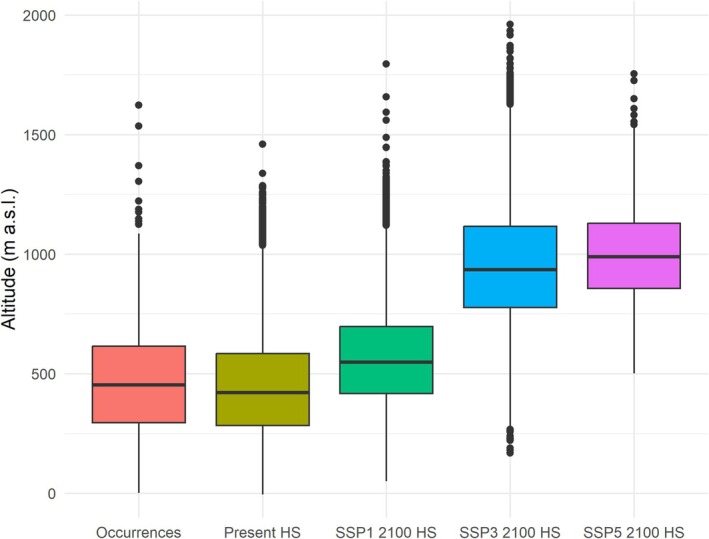
Boxplots comparing the distribution of altitude values for present suitable habitat, species occurrence points, and projected future suitable habitat under different climate change scenarios (SSP1, SSP3, and SSP5). These visualizations demonstrate the upward shift in altitude with increasing greenhouse gas emissions. Summary statistics are reported in Appendix A: Table A4.

Under the optimistic SSP1 scenario, high‐suitability areas initially shrink by 2070 but recover slightly by 2100, reaching 24,100 km^2^, with a loss of approximately 32% of the current total (Table 3). This fluctuation reflects the scenario's assumption of stringent climate mitigation measures, resulting in a long‐term stabilization of temperatures and precipitation patterns. However, the areas with recovered suitability are not always spatially aligned with current habitats, shifting slightly to the north and to higher altitudes (Figures 4c and 6).

**TABLE 3:** Total extent of high‐suitability areas for each scenario.

Period		SSP1	SSP3	SSP5
Present	35,273 km^2^			
2040		27,391 km^2^	27,471 km^2^	26,279 km^2^
2070		21,881 km^2^	17,535 km^2^	20,578 km^2^
2100		24,100 km^2^	15,121 km^2^	251 km^2^

In contrast, the intermediate SSP3 scenario predicts a progressive decline in high‐suitability areas, culminating in a reduction from 35,273 to 15,121 km^2^ by 2100, a 55% loss compared to the present (Table 3). Central Italian regions like Latium and Tuscany, which currently host the densest occurrences, lose much of their suitability, while areas with high suitability, such as those present in the Ligurian Apennines and Calabrian Pollino and Sila massifs, shift to higher altitudes (Figures 4f and 6). Despite the absence of present occurrences, the Rhône River basin in France is suggestive of a great potential for high ecological suitability at northern latitudes.

The SSP5 scenario, characterized by unmitigated emissions and rapid warming, presents the most dramatic habitat loss, with high‐suitability areas dwindling to just 251 km^2^ by 2100, a 99% reduction compared to the present (Table 3). These remaining suitable habitats are confined almost exclusively to the coastal area between northern Tuscany and the France‐Italy border, highlighting the catastrophic impact of this high‐emission scenario. In this scenario, the Rhône River basin no longer offers suitable habitat (Figures 4i and 5).

**FIGURE A5:**
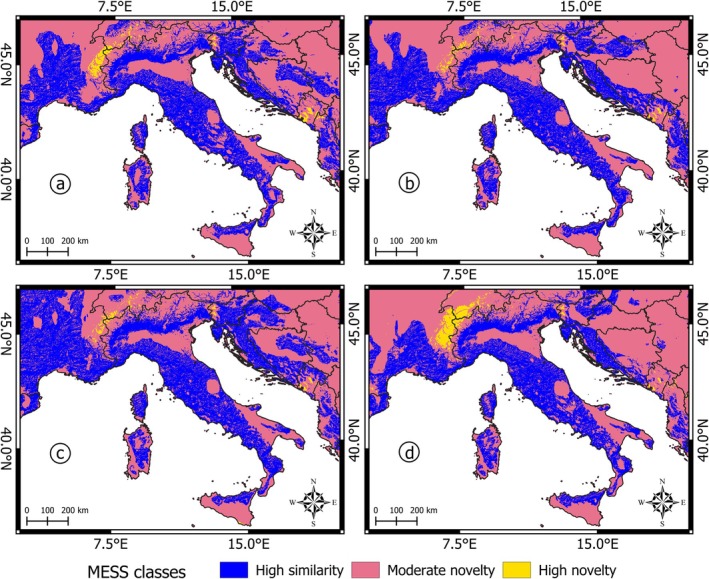
MESS analysis showing areas of model extrapolation and prediction reliability for (a) current conditions, (b) predicted conditions under SSP1 by 2100, (c) predicted conditions under SSP3 by 2100, and (d) predicted conditions under SSP5 by 2100. High similarity areas (values > 0) indicate conditions within or close to the training range, resulting in reliable predictions. Moderate novelty areas (−50 to 0) denote reduced prediction reliability. High novelty areas (values < −50) represent extreme dissimilarity from training data, leading to highly uncertain predictions.

In Appendix A: Figure A5, are presented the results of the MESS analysis, which was used to assess the reliability of the present and 2100 future projections.

## 4 Discussion

The geographical distribution and evolutionary history of *Q. crenata* present several unanswered questions, making it a particularly interesting case study of forest biodiversity in the Euro‐Mediterranean region. This work fills the existing knowledge gap on the species' distribution, integrating previous cluttered data with new information gathered through direct field observations and in‐depth literature analysis. Hence, punctual distribution and modeled ecological niche of *Q. crenata* are put forward as crucial steps to foster future conservation programs and drive novel studies aimed at clarifying the species' identity, origin, and standing genetic diversity.

However, MaxEnt‐based models are obviously not devoid of some limitations. The bioclimatic, edaphic, and topographic variables generally used may not encompass all factors influencing a species' distribution. For instance, species interactions, pathogens, and human activities (such as indiscriminate logging opposed to historical preservation of impressive individuals) could be significant as well, but the complexity of these factors, combined with a lack of spatially explicit data, prevents their incorporation into current modeling approaches. Despite these limitations, our study represents the first application of a distribution model to estimate the potential range of a European sporadically distributed tree species. The relevant number of the collected records was essential in ensuring a well‐supported model and making the obtained results extremely valuable to suggest adequate conservation strategies. To these aims, the crucial role of field surveys, combined with comprehensive data mining and verification in herbaria and publicly accessible online data is remarked, especially for yet poorly known and sparsely distributed species. Our future projections rely on predictions from a single Earth System Model, MPI‐ESM1.2‐HR. Although this model was chosen for its high resolution, which is particularly beneficial for capturing the climate heterogeneity of the Mediterranean region, relying on a single model implies that our analysis does not fully evaluate the range of uncertainty associated with future climate projections across different climate models. Consequently, considering both the reliance on a single Earth System Model and the inherent uncertainty of making long‐term predictions at a 1 km resolution, the future projections presented here should be interpreted as a first attempt to describe the general trends in habitat suitability and range shifts under the considered SSP scenarios, rather than precise spatial predictions. Nevertheless, we are confident that our approach can serve as a useful guideline for similar studies on other sporadic or scattered species deserving protection, at both continental and national scales, as well as a basis for future works on *Q. crenata* .

### 4.1 Current Distribution and Conservation Status

The resulting 923 occurrences significantly increase the available knowledge on the actual distribution of *Q. crenata* and accurately delineate its current range. However, total documentation accounts for an alarming count of < 2000 individuals (Frankham 2022; Hoban et al. 2023), a key factor supporting its categorization as a rare species. According to the criteria recently synthesized by Crisfield et al. (2024), *Q. crenata* rarity is further evident in the extremely low individual numbers across its distribution (low abundance), the scattered and sporadic occurrences across its geographic extent (low occupancy), and the limited overall range extension. Furthermore, its rarity is compounded by high exposure to various contingencies, including significant predicted habitat losses under climate change scenarios, yet undetermined species interactions, and substantial anthropogenic pressure, all contributing to increase the species vulnerability.

Sparse groups and isolated plants of *Q. crenata* currently extend from the central and northern Apennines to the coasts of the Tyrrhenian Sea, passing through the Ligurian Apennines to the French Riviera. Even fewer, scattered individuals occur in the Italian Prealps, along the Adriatic coasts of Italy, Slovenia, and Croatia, and in southern Italy. While confirming previous generic maps (Jalas and Suominen 1976; Sicily deleted by Brullo et al. 1999), our results significantly highlight Latium and Tuscany as the core regions of *Q. crenata* distribution and further provide a fundamental baseline for assessing its current conservation status and identifying key areas for protection. In this regard, the updated distribution map reveals that approximately 36% of *Q. crenata* occurrences are located outside national or regional protection and/or outside Natura 2000 sites (protection status details available in the dataset description at https://zenodo.org/records/14945096), emphasizing the need for increased conservation efforts. This urgency is further stressed by the varying levels of protection conferred to the species across its range. Although *Q. crenata* is officially recognized and protected in seven Italian regions (Piedmont, Lombardy, Emilia‐Romagna, Tuscany, Umbria, Marche, Molise), with a total of 346 occurrences, and a further 20 individuals are under strict protection regime, listed as “Monumental trees” by Italian Minister of Agriculture and Forests (https://www.politicheagricole.it; updated to Nov. 2024), still a significant proportion of the Italian records (including those from Latium region, Liguria and the rare north‐eastern and southern individuals) remains without protection. On the other hand, all French and the few Slovenian occurrences are nationally classified as Vulnerable or Threatened and consequently protected. However, the species is not currently included in any conservation list for Croatia, despite the documented rare occurrences. The low numbers of all extant *Q. crenata* individuals and the disparity in conservation status across countries and regions (Table 4) clearly urge a coordinated and comprehensive conservation strategy at the international level.

**TABLE 4:** Protection status of *Quercus crenata*.

Country	Protection status	Non‐protected occurrences	Notes
Italy	Officially protected in seven regions (Piedmont, Lombardy, Emilia‐Romagna, Tuscany, Umbria, Marche, and Molise); individuals under strict “Monumental Tree” protection	325/867 (37%)	Significant portion of Italian records (Latium, Liguria, rare Northeastern/Southern individuals) not protected
France	Nationally classified as Vulnerable/Threatened	None	All occurrences protected
Slovenia	Nationally classified as Vulnerable/Threatened	None	All occurrences protected
Croatia	Not currently listed	3/3 (100%)	No current protection
Overall	Outside national or regional protection and/or outside Natura 2000 sites	328/923 (36%)	

### 4.2 Ecology of *Q. crenata* and Potential Species Range

The key environmental factors influencing *Q. crenata* distribution revealed by the model point toward a strong adaptation to Mediterranean climatic conditions. The importance of precipitation seasonality and the preference for moderate temperature fluctuations suggest a species thriving in a mild Mediterranean climate, where seasonal variability is present, but droughts are not severe. The apparent need for sufficient annual precipitation indicates moderate to high water requirements and a potential vulnerability to water scarcity, although it is adaptable to a variety of soil types. These results align well with previous ecological descriptions, which provided a partial understanding of *Q. crenata* ecology at a local scale (Mercurio 1985; Cresta and Salvidio 1991; Armiraglio et al. 2003), particularly regarding its water requirements and soil tolerance. Furthermore, our model is in agreement with the general pattern produced by Denk et al. (2023) who, even if based on a limited number of occurrences (84) retrieved from GBIF database (https://www.gbif.org/), characterized *Q. crenata* as a sub‐Mediterranean member of the Temperate Broadleaf and Mixed Forest biome with extension into the Mediterranean Forests, Woodlands and Scrublands biome (Olson et al. 2001), with a Köppen–Geiger climate profile (Kottek et al. 2006; Peel et al. 2007) comprising Warm temperate summer‐dry to fully humid climates, with warm or hot summers (Csa, with extensions into Csb, Cfb).

Based on our model, the potential range of *Q. crenata* appears to extend beyond its currently known distribution, including vast portions of South Italy (northern Sicily, much of the territories of Calabria, Campania, and Molise), and the Rhone basin in the French regions of Rhône‐Alpes and Languedoc‐Roussillon. Although the poorly recorded species' presence in these high‐suitability areas matches the scarce historical and present evidence, and provided the species has not been progressively decimated by men, future targeted searches may reveal if the species is actually thriving in inaccessible areas or if it is being overlooked by foresters and botanists, thereby leading to an improved assessment of the species' distribution and ecology. However, although desirable, a partial increase in the number of records would hardly be decisive in reversing the critical low census size detected in this study. In this regard, it is relevant to note that Brullo et al. (1999) excluded *Q. crenata* from the Sicilian flora and reported instead the presence of a highly similar *taxon*, *Q. fontantesii* Guss., occurring as small groups or isolated plants in the submontane belt of the north Sicilian Mountain systems (between 200 and 800 m a.s.l.), and considered the stabilized hybrid between *Q. suber* and *Q. gussonei* (Borzì) Brullo. Both *Q. fontanesii* and *Q. gussonei* are Sicilian endemic oaks currently accepted in main Italian and online World floras (Pignatti et al. 2017; https://www.ipni.org/; https://powo.science.kew.org/). However, *Q. gussonei* appears as a mere thermophilus form of *Q. cerris* and *Q. fontanesii* is morphologically indistinguishable from *Q. crenata* (Schicchi et al. 2000; Cristofolini et al. 2017). The potentially suitable areas of *Q. crenata* we identified in Sicily perfectly overlap those of *Q. fontanesii*, thereby pointing to a close correspondence between the two *taxa*. A thorough geno‐taxonomic investigation would therefore be needed to disentangle such a questionable nomenclature and eventually expand the current distribution of *Q. crenata* to Sicily.

### 4.3 “Tyrrhenian” Affinity and Evolutionary Questions

Our data suggests a possible “Tyrrhenian” affinity of the *Q. crenata* distribution, given that most of the high‐suitability areas are located along the Tyrrhenian coast or in close‐by regions. This could reflect a historical pattern of the species establishment in suitable (mesic) habitats and survival through times, likely determined by the peculiar geo‐morphological and climate changes of the Euro‐Mediterranean area since the Miocene and in the Pliocene, when a mosaic of different routes and local/regional ecological conditions occurred (Suc 1984; Krijgsman 2002; Magri 2010), followed by persistence in local Pleistocene refugia (Médail and Diadema 2009; for further evidence of Italian Glacial refugia of oaks along the Tyrrhenian coast see also Di Pietro et al. 2024). At the same time, the concentration of *Q. crenata* occurrences within the Tyrrhenian regions, also characterized by overlapping ranges of its presumed parental species ( *Q. cerris* and *Q. suber* ), lends support to the hypothesis of a recent hybrid origin. However, recent phylogenomic studies and the presence of *Q. crenata* individuals in areas where *Q. suber* is absent reinforce questions about the complexity of the evolutionary processes that led to the formation of this *taxon* and support the hypothesis of an autonomous, today relict species among West Eurasian oaks, possibly coexisting with few, morphologically indistinguishable, hybrid individuals (Cristofolini and Crema 2005; Denk et al. 2023). Resolving the complex evolutionary puzzle of *Q. crenata* is not easy, given the current lack of diagnostic standard molecular markers (Conte et al. 2007; Bagnoli et al. 2016), but it would be critical to understand the species' adaptability to future environmental changes. As an ancient evolutionary leftover, it may possess deep‐time genetic legacies (“trait conservatism”; Cavender‐Bares et al. 2016), possibly making it preadapted to face multifaceted ecological changes. However, the possibility of reduced genetic diversity and drifting, due to its extremely low population numbers, should be carefully evaluated. Conversely, as a (either recent or stabilized) hybrid *taxon*, it may possess increased genetic diversity and be favored by the transfer of adaptive alleles from the genetic backgrounds of both parents, enhancing local adaptation and allowing it to respond quickly to selective pressures (Rieseberg and Willis 2007; Taylor et al. 2015). In this case, the roles of climate, landscape, demography, and genetic diversity of the parental species should be adequately considered (Bolte et al. 2024). Our results show that *Q. crenata* has some phenotypic plasticity, despite the extremely low population numbers and the current lack of population genetic studies. Combining the here assessed species ecology and demographic data with novel studies on the species evolutionary history and the geographic structure of its genetic diversity would be essential to comprehend how *Q. crenata* will tackle future environmental changes. As an intriguing add‐on, hindcasting *Q. crenata* distribution since the last Interglacial could reveal whether it experienced range stability or significant shifts and eventually indicate larger range overlaps with the presumed parental species (Vessella et al. 2015; Bagnoli et al. 2016).

### 4.4 Climate Change Impacts and Conservation Efforts

Future projections regarding changes in high‐suitability areas suggest that rising temperatures and changes in rainfall patterns could significantly impact *Q. crenata* habitats, with varying degrees of severity depending on future greenhouse gas emission scenarios. Under the more optimistic SSP1 scenario, while initial habitat shrinkage is expected, some recovery is possible by the end of the century, though the location of suitable areas may shift, potentially complicating conservation. The moderate SSP3 scenario projects a substantial decline in suitable habitat, particularly areas with currently numerous occurrences, with remaining suitable areas shifting to higher altitudes and more northerly latitudes, including regions where the species is not currently found. The most severe SSP5 scenario paints a dire picture, with a near‐total loss of suitable habitat by the end of the century, leaving only small, fragmented refugia. These projections underscore the critical need for effective climate change mitigation strategies to preserve suitable habitat for this species. The coastal area between northern Tuscany and the France‐Italy border could emerge as a priority area for the conservation of *Q. crenata* . Therefore, for the future, an in‐depth assessment of the possible migration routes and strategies necessary to facilitate the movement of the species is crucial, along with a thorough implementation of current conservation measures.

In addition, if the hybrid origin hypothesis for *Q. crenata* is confirmed, comparing its future distribution to that of the putative parent species ( *Q. cerris* and *Q. suber* ) will be critical as well. Recent studies showed that under intermediate emission scenarios *Q. cerris* is projected to expand northward and to higher altitudes, potentially increasing its presence in Central European regions, while its distribution in Italy may contract (Illés and Móricz 2022). *Quercus suber* , conversely, is expected to experience moderate range contraction without significant shifts (Vessella et al. 2017). Despite some contraction in areas suitable for *Q. crenata* , the continued presence of both parent species in close‐by areas may thus help maintain some hybridization, possibly allowing demographic renewal. However, under extreme emission scenarios, *Q. cerris* is likely to experience greater area losses in Italy, particularly at lower altitudes (Illés and Móricz 2022), while *Q. suber* is expected to face severe range reduction, with high‐suitability areas shrinking to a few isolated refugia (Vessella et al. 2017). This near‐total loss of *Q. suber* suitable habitats across much of its range and the consequent limitation of genetic exchange with *Q. cerris* therefore raises important concerns about *Q. crenata* 's future presence. The extinction risk could be further exacerbated by the restricted ecologically suitable areas for *Q. crenata* , which are projected to become extremely fragmented.

In this scenario, recommendations for the sustainable conservation and management of *Q. crenata* should certainly consider a prompt assessment of the species' status at the international level and involve steering policy and actions to search and catalog, monitor, and protect all *Q. crenata* individuals across its range. We emphasize the conservation priority of all present occurrences and call for future research aimed at refining the current distribution, particularly in the southern regions, and resolving all taxonomic ambiguities. In our opinion, a more accurate and complete picture of the present‐day distribution is the crucial starting point for informing effective conservation actions. Embracing the recent European Nature Restoration Law (http://data.europa.eu/eli/reg/2024/1991/oj), a key element of the EU Biodiversity Strategy for 2030 (European Commission 2021), coordinated international plans should therefore pursue maintenance, connection, restoration, and possibly an increase of the forest habitats where *Q. crenata* (and the putative parental species, *Q. cerris* and *Q. suber* ) grows. To enlarge *Q. crenata* population quantity, germplasm banking for ex situ conservation programs in botanic gardens, arboreta, or other research institutions and plantations addressing afforestation/reforestation initiatives and tree planting in urban and peri‐urban contexts could play an important role as well. Finally, studies on allelic diversity and genetic structure of *Q. crenata* would be of utmost importance. Besides revealing the efficacy of gene flow, which is especially critical for the long‐term survival of sporadic tree species, these studies could allow the identification of particularly vulnerable individual/populations or those with higher adaptive potential.

## 5 Conclusion

In this work, we provide an exhaustive assessment of the actual geographical distribution of *Q. crenata* , describe its ecology, and explore the consequences of climate change on its potential habitats to recommend conservation actions. *Q. crenata* mostly thrives along west central and northern regions of Italy, with scattered occurrences in confining regions, generally outside protected areas. As a member of moderately mesic forests and open habitats, with strong adaptation to Mediterranean climatic conditions, the species ecology is primarily determined by sufficient precipitation and moderate temperature fluctuations; both features are projected to be highly impacted by global change in the next decades. Furthermore, the overall count of the recorded individuals suggests an alarming census size of no more than 2000 adult trees, highlighting serious risks of species extinction and/or inbreeding depression with loss of genetic diversity and adaptive potential. Promoting individual and stand protection, monitoring health and reproduction, appears thus mandatory; at the same time, artificial plantations could improve genetic connectivity, a fundamental issue especially for species having small, scattered populations and a fragmented distribution.

In Italy, all gathered *Q. crenata* records become gradually rarer moving southwards of the core distribution range, despite the potential ecological suitability we estimated in some regions. One additional, major outcome of our results is therefore to stress future searches in all those suitable areas, to definitely assess the species range and individual numbers, with a crucial impact on the species' conservation issues. In this view, we further call for deeper analyses to understand the true nature and origin of this oak and possibly resolve all detrimental cases with a controversial nomenclature.

## Author Contributions

**Giuseppe Antonelli:** conceptualization (equal), data curation (equal), formal analysis (lead), funding acquisition (equal), investigation (equal), methodology (lead), project administration (supporting), resources (equal), software (equal), supervision (equal), validation (equal), visualization (equal), writing – original draft (equal), writing – review and editing (equal). **Giuseppe Puddu:** conceptualization (equal), data curation (equal), formal analysis (equal), funding acquisition (equal), investigation (lead), methodology (equal), project administration (supporting), resources (equal), software (equal), supervision (equal), validation (equal), visualization (equal), writing – original draft (equal), writing – review and editing (equal). **Chiara Cipollini:** conceptualization (supporting), data curation (equal), formal analysis (supporting), funding acquisition (supporting), investigation (supporting), methodology (supporting), project administration (supporting), resources (equal), software (supporting), supervision (supporting), validation (supporting), visualization (supporting), writing – original draft (equal), writing – review and editing (supporting). **Gianluca Sabatini:** conceptualization (supporting), data curation (equal), formal analysis (supporting), funding acquisition (supporting), investigation (supporting), methodology (supporting), project administration (supporting), resources (supporting), software (supporting), supervision (supporting), validation (supporting), visualization (supporting), writing – original draft (equal), writing – review and editing (equal). **Maurizio Conticelli:** conceptualization (supporting), data curation (supporting), formal analysis (supporting), funding acquisition (supporting), investigation (supporting), methodology (supporting), project administration (supporting), resources (supporting), software (supporting), supervision (supporting), validation (supporting), visualization (supporting), writing – original draft (equal), writing – review and editing (equal). **Marco Cosimo Simeone:** conceptualization (equal), data curation (equal), formal analysis (supporting), funding acquisition (equal), investigation (supporting), methodology (supporting), project administration (lead), resources (equal), software (supporting), supervision (equal), validation (equal), visualization (equal), writing – original draft (equal), writing – review and editing (equal).

## Conflicts of Interest

The authors declare no conflicts of interest.

## Figures and Tables

**FIGURE A6:**
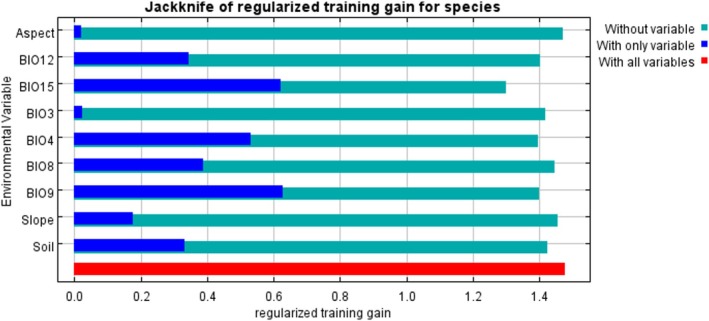
Jackknife test of variable importance. BIO9 exhibited the highest gain when used in isolation, indicating its strong individual predictive power. BIO15 showed the largest decrease in gain upon omission, suggesting that it contributes unique information not captured by other variables.

## Data Availability

The detailed list of all the *Q. crenata* occurrences, relevant R scripts, and the environmental variable layers used for the model is available at https://zenodo.org/records/14945096. The spatial prediction layers, representing predicted habitat suitability for current and future conditions as shown in Figures 3, 4, 5, are available upon request. All other relevant information is included within the manuscript.

## Appendix A

**TABLE A1:** Description of the environmental layers.

Variable	Description		Units of measurement
BIO1	Mean annual air temperature		°C
BIO2	Mean diurnal air temperature range		°C
BIO3	Isothermality		°C
BIO4	Temperature seasonality		°C/100
BIO5	Mean daily maximum air temperature of the warmest month		°C
BIO6	Mean daily minimum air temperature of the coldest month		°C
BIO7	Annual range of air temperature		°C
BIO8	Mean daily mean air temperatures of the wettest quarter		°C
BIO9	Mean daily mean air temperatures of the driest quarter		°C
BIO10	Mean daily mean air temperatures of the warmest quarter		°C
BIO11	Mean daily mean air temperatures of the coldest quarter		°C
BIO12	Annual precipitation amount		kg m^−2^ year^−1^
BIO13	Precipitation amount of the wettest month		kg m^−2^ month^−1^
BIO14	Precipitation amount of the driest month		kg m^−2^ month^−1^
BIO15	Precipitation seasonality		kg m^−2^
BIO16	Mean monthly precipitation amount of the wettest quarter		kg m^−2^ month^−1^
BIO17	Mean monthly precipitation amount of the driest quarter		kg m^−2^ month^−1^
BIO18	Mean monthly precipitation amount of the warmest quarter		kg m^−2^ month^−1^
BIO19	Mean monthly precipitation amount of the coldest quarter		kg m^−2^ month^−1^
Elevation	Elevation		m a.s.l.
Aspect	Aspect, reclassed in eight classes		
	*ID*	*Degrees*	
	0	From 0° N to 45°	
	1	From 45° to 90°	
	2	From 90° to 135°	
	3	From 135° to 180°	
	4	From 180° to 225°	
	5	From 225° to 270°	
	6	From 270° to 315°	
	7	From 315° to 360°	
Slope	Slope		
Soil	WRB soil classification classes		
	*ID*	*Soil type*	
	1	Acrisols	
	2	Alisols	
	3	Andosols	
	4	Arenosols	
	5	Anthrosols	
	6	Chernozems	
	7	Calcisols	
	8	Cambisols	
	9	Cryosols	
	10	Fluvisols	
	11	Ferralsols	
	12	Glaciers	
	13	Gleysols	
	14	Gypsisols	
	15	Histosols	
	16	Islands	
	17	Kastanozems	
	18	Leptosols	
	19	Luvisols	
	20	Lixisols	
	21	Nitisols	
	22	Phaeozems	
	23	Planosols	
	24	Plinthosols	
	25	Podzols	
	26	Regosols	
	27	Retisols	
	28	Solonchaks	
	29	Solonetz	
	30	Stagnosols	
	31	Technosols	
	32	Umbrisols	
	33	Vertisols	
	34	Open water	
	35	No data	

**TABLE A2:** ODMAP (Overview, Data, Model, Assessment, Prediction) protocol describing the workflow used to model the probability of occurrence of *Quercus crenata* Lam.

Overview	
Authorship	Author contribution: provided in the main text Contact: provided in the main text
Model objective	Model objective: predict potential current and future distribution of *Quercus crenata* Lam. Target output: a map of current distribution with four levels of presence probability; nine maps of future distribution showing the extent of high‐suitability areas under SSP1, SSP3, and SSP5 each for 2040, 2070, and 2100; three maps for 2100 highlighting gain and loss in high‐suitability areas extent
Focal taxon	*Q. crenata* Lam.
Location	Southern Europe
Scale of analysis	Spatial extent: −75,000, 1,481,000, 4,060,000, 5,225,000 (xmin, xmax, ymin, ymax) [WGS 84/UTM 32N; EPSG:32632] Spatial resolution: 1 km Temporal extent: 1981–2010 (prediction: 2011–2100) Temporal resolution: 30 years Boundary: rectangle
Biodiversity data	Observation sources: field samplings, literature surveys, online databases Response data type: presence‐only
Predictors	Rasters describing bioclimatic, topographic and edaphic conditions.
Hypotheses	Due to limited ecological knowledge of *Q. crenata*, broad hypotheses were adopted, focusing on precipitation to capture water availability, seasonality to distinguish climatic patterns, and soil types to reflect its adaptability to diverse environments
Assumptions	The species is in equilibrium with its environment and its ecological niche is stable
Algorithms	Modeling techniques: MaxEnt algorithm (Phillips et al. 2006) within an R environment (dismo::maxent function) Model complexity: since overfitting can indicate overly complex models, the absolute difference between training and validation AUC (AUC_diff_; Warren and Seifert 2011) was used as a proxy, with smaller values suggesting better generalization and reduced model complexity.
Workflow	To optimize MaxEnt model parameters and reduce overfitting, 50 candidate models were tested by varying feature class configurations (L, LQ, LQH, LQHP, and LQHPT) and regularization multipliers (0.5–5.0, in 0.5 increments). To minimize spatial autocorrelation (Wenger and Olden 2012; Radosavljevic and Anderson 2014), the 50 candidates were evaluated using “block” cross‐validation within the ENMeval::ENMevaluate function in R. Selection metrics included Akaike Information Criterion correction (AICc; Burnham and Anderson 1998), AUC_diff_, and Continuous Boyce Index (CBI; Hirzel et al. 2006). The best‐performing model was chosen by prioritizing a balance of model fit (minimizing AICc and AUC_diff_) and predictive performance (maximizing CBI), while also considering the ecological interpretability of the response curves (Elith et al. 2006)
Software	Software: R version 4.3.3 (2024‐03‐01) Code and data availability: https://zenodo.org/records/14945096 Packages versions: car 3.1.3, corrplot 0.92, dismo 1.3‐14, ecospat 4.0.0, ENMeval 2.0.4, ggplot2 3.5.1, openxlsx 4.2.6.1, raster 3.6‐26, rJava 1.0‐11, terra 1.7‐71
Data	
Biodiversity data	Taxonomic reference system: World Flora Online (WFO; worldfloraonline.org/) Ecological level: species Data sources: field samplings, literature surveys, online databases Sampling design: opportunistic sampling Sample size: 705 occurrences, one for each 1 km cell Background data: 20,000 randomly generated background points
Data partitioning	“block” cross‐validation within the ENMeval::ENMevaluate function in R
Predictor variables	Predictor variables: climatic conditions were represented by 19 bioclimatic variables; topographic variables included altitude, slope, and aspect; edaphic factors were described by soil types based on the World Reference Base for Soil Resources. A full description of each variable is provided in Table A1 Data sources: climatic conditions, computed for the period 1981–2010, were obtained from the CHELSA repository (Karger et al. 2017), version 2.1 (Karger et al. 2021), at a native resolution of 30 arc‐sec. The Digital Elevation Model was sourced from ESA Copernicus GLO‐90 v2023.1 (ESA, 2023) at a native resolution of 90 m through the OpenTopography repository; slope and aspect were calculated using the terra::terrain function in R. Soil data were retrieved from the Harmonized World Soil Database v2.01 (FAO and IIASA 2023) at a native resolution of 30 arc‐sec Spatial extent: all variables were cropped to the rectangular extent indicated above. Spatial resolution: all variables were resampled to a 1 km resolution using the terra::resample function in R, with cubic interpolation for continuous variables and nearest neighbor for the categorical variables Coordinate reference system: WGS 84/UTM zone 32N Temporal extent: bioclimatic variables 1981–2010
Transfer data	Data sources: CHELSA repository (Karger et al. 2017), version 2.1 (Karger et al. 2021) future scenarios Spatial extent: Southern Europe Spatial resolution: 1 km Temporal extent: current 1981–2010, future 2011–2100 Models and scenarios: MPI‐ESM1.2‐HR (Gutjahr et al. 2019) Quantification of novelty: assessed by calculating the Multivariate Environmental Similarity Surface (MESS; Elith et al. 2010) using the ecospat::mess function in R
Model	
Multicollinearity	An iterative variable selection process was conducted using Pearson's correlation coefficient. A correlation matrix for all 23 environmental variables (3 topographic, 1 soil, and 19 bioclimatic; Figure A1) was calculated using the corrplot::cor function in R, where pairs of variables with a correlation coefficient (|*r*|) greater than 0.7 (Dormann et al. 2013) were identified as highly correlated. To reduce multicollinearity, the variable with the highest average correlation to others was iteratively removed, and the process was repeated until all remaining variables had a correlation coefficient below the threshold (Figure A2). During this process, BIO12 (annual precipitation), BIO4 (temperature seasonality), and BIO15 (precipitation seasonality) were “locked” due to their assumed ecological relevance. Additionally, a Variance Inflation Factor (VIF) analysis was conducted using the car::vif function in R, with a threshold of 5, indicating low correlation among predictors (James et al. 2013; Figure A3)
Model settings	The best‐performing model (“fc.LQHP_rm.2”, Table A3) included linear, quadratic, product, and hinge feature classes, with a regularization multiplier value of 2
Model estimates	Assessment of variable importance: percent contribution (increase in regularized gain) and variable importance (drop in training AUC after permutation), as provided by MaxEnt output (main text, Table 1). Additionally, a jackknife analysis (Figure A6) was performed
Threshold selection	Details on threshold selection: for the present projection threshold selected were Minimum Training Presence (MTP, resulted equal to 0.0078), 10th Percentile Training Presence (P10, equal to 0.1746), and a fixed value of 1–1/e ≈ 0.632 (Phillips et al. 2017). For future predictions, a binary classification was used, with a single threshold of 0.632
Assessment	
Performance statistics	Performance on training data: AUC_train_ = 0.938; CBI_train_ = 0.989 Performance on validation data: AUC_val_ = 0.912 ± 0.046; CBI_val_ = 0.876 ± 0.140
Plausibility check	Response curves were examined in the best performing model selection to ensure ecological relevance (Elith et al. 2006; Figure A4)
Prediction	
Prediction output	Prediction output: the current and future predictions from the best‐performing model are provided in the main text (Figures 3, 4, 5) Prediction unit: probability values ranging from 0 to 1 (cloglog output, Phillips et al. 2017); following common practice in species distribution modeling these probabilities are interpreted as relative habitat suitability (Guisan et al. 2017)
Uncertainty quantification	Scenario uncertainty: not evaluated, since for future projection only the MPI‐ESM1.2‐HR (Gutjahr et al. 2019) was used. Novel environments: extrapolation and prediction reliability were evaluated using a MESS analysis (Figure A5) under present and future (SSP1, SSP3, and SSP5 by 2100) conditions

**TABLE A3:** Evaluation metrics for the 20 best‐performing MaxEnt candidate models, ordered by AICc. Metrics include AUC for the validation set (AUC_val_), the difference between training and validation AUC (AUC_diff_), and the Continuous Boyce Index for the validation set (CBI_val_). The selected model, “fc.LQHP_rm.2,” is shown in bold.

Candidate model	Features	RM	Average AUC_val_	Std.dev. AUC_val_	Average AUC_diff_	Std.dev. AUC_diff_	Average CBI_val_	Std. dev. CBI_val_	ΔAICc
fc.LQHPT_rm.1	LQHPT	1	0.910	0.045	0.054	0.036	0.718	0.342	0.000
fc.LQHPT_rm.1.5	LQHPT	1.5	0.911	0.044	0.051	0.032	0.776	0.232	45.220
fc.LQHPT_rm.0.5	LQHPT	0.5	0.909	0.044	0.057	0.042	0.711	0.422	46.095
fc.LQHP_rm.0.5	LQHP	0.5	0.917	0.042	0.048	0.027	0.821	0.182	91.400
fc.LQHPT_rm.2	LQHPT	2	0.911	0.044	0.049	0.033	0.849	0.146	93.302
fc.LQHP_rm.1	LQHP	1	0.914	0.044	0.048	0.029	0.867	0.147	110.789
fc.LQHPT_rm.2.5	LQHPT	2.5	0.910	0.045	0.049	0.034	0.843	0.157	133.142
fc.LQHPT_rm.3	LQHPT	3	0.908	0.046	0.049	0.036	0.851	0.146	143.943
**fc.LQHP_rm.2**	**LQHP**	**2**	**0.912**	**0.046**	**0.047**	**0.032**	**0.876**	**0.140**	**145.130**
fc.LQHPT_rm.3.5	LQHPT	3.5	0.908	0.046	0.048	0.037	0.857	0.128	147.884
fc.LQHPT_rm.4	LQHPT	4	0.907	0.047	0.048	0.039	0.867	0.105	168.668
fc.LQHP_rm.1.5	LQHP	1.5	0.913	0.045	0.048	0.031	0.863	0.165	173.380
fc.LQH_rm.1	LQH	1	0.912	0.046	0.049	0.030	0.856	0.163	176.556
fc.LQH_rm.1.5	LQH	1.5	0.911	0.046	0.048	0.033	0.870	0.169	188.912
fc.LQH_rm.0.5	LQH	0.5	0.913	0.043	0.050	0.028	0.851	0.149	189.389
fc.LQHP_rm.2.5	LQHP	2.5	0.911	0.046	0.047	0.034	0.894	0.117	191.674
fc.LQH_rm.2	LQH	2	0.910	0.047	0.048	0.034	0.890	0.134	212.146
fc.LQHP_rm.3	LQHP	3	0.910	0.047	0.047	0.036	0.911	0.100	216.737
fc.LQHP_rm.3.5	LQHP	3.5	0.908	0.048	0.048	0.036	0.916	0.080	218.739
fc.LQHPT_rm.5	LQHPT	5	0.906	0.047	0.049	0.040	0.873	0.078	219.142

**TABLE A4:** Comparison of mean altitude for present high‐suitability (HS) areas (used as a reference) against species occurrence points and projected future (by 2100) HS areas. Altitude values were assigned to HS pixels within present and future projections using the “Altitude” variable layer. Species occurrences were observed at a higher mean altitude compared to present HS areas. This difference likely reflects the distinct nature of the underlying data: Occurrence data includes all recorded observations, potentially encompassing marginal habitats and high‐elevation outliers, whereas present HS areas are defined based on optimal ecological conditions. Projected future scenarios show an upslope shift in mean altitude, with progressively larger shifts from present HS to SSP1, SSP3, and then SSP5 HS areas. Welch's *t*‐tests were used to assess the statistical significance of the difference between each future scenario and present HS.

Data source	Sample size	Mean altitude	Difference from Present HS mean altitude	Mean altitude std.dev.	*p*‐value
Occurrences	923	473.2 m	+27.4 m	245.6 m	0.00085
Present HS	35,273	445.8 m	—	220.2 m	—
SSP1 HS	26,279	565.6 m	+119.8 m	212.1 m	< 2.2e‐16
SSP3 HS	20,578	953.3 m	+507.5 m	247.3 m	< 2.2e‐16
SSP5 HS	251	1009.1 m	+563.3 m	270.3 m	< 2.2e‐16

*Note:* Given the substantial differences in sample sizes across all scenarios, a Kruskal–Wallis test was also performed. The results (*χ* ^2^ = 28,626; df = 4; *p*‐value < 2.2e‐16) confirmed a statistically significant difference in mean altitude among all scenarios.
